# Liver Transplantation as a Treatment for Unresectable Hepatic Adenoma in a Patient With Abernethy Syndrome

**DOI:** 10.7759/cureus.60683

**Published:** 2024-05-20

**Authors:** Shreeja Patel, Dane Thompson, Mohamed Sharshar, James M Crawford, Nabil Dagher, Ahmed E Fahmy

**Affiliations:** 1 Division of Transplant Surgery, Donald and Barbara Zucker School of Medicine at Hofstra/Northwell, New York, USA; 2 Department of Surgery, Donald and Barbara Zucker School of Medicine at Hofstra/Northwell, New York, USA; 3 Division of Transplant Surgery, Department of Surgery, Northwell Health, New York, USA; 4 Department of Pathology and Laboratory Medicine, Northwell Health, New York, USA; 5 Department of Pathology and Laboratory Medicine, Donald and Barbara Zucker School of Medicine at Hofstra/Northwell, New York, USA

**Keywords:** portosystemic shunt, congenital portosystemic shunt, hepatocellular adenoma, liver transplant physician, congenital malformation, liver transplant, unresectable adenoma, liver adenoma, transplantation, abernethy syndrome

## Abstract

Abernethy syndrome is a rare congenital anomaly characterized by an intrahepatic or extrahepatic portosystemic shunt. Most patients are asymptomatic; however, due to the alteration in, or lack of, a portovenous flow, patients with Abernethy syndrome are at high risk of developing sequelae of liver failure. Once these complications develop, the only definitive treatment is transplantation. Patients with Abernethy syndrome are also at a higher risk of developing benign and malignant liver lesions, including hepatic adenomas. Here, we describe the first case of deceased donor liver transplantation as a treatment for a patient with type 1 Abernethy syndrome complicated by large, unresectable hepatic adenoma, found to have focal hepatocellular carcinoma on pathologic examination. Our male patient was found to have elevated liver enzymes at age 33, during a routine outpatient medical appointment. Despite being asymptomatic, his history of prior liver resection prompted CT imaging, which revealed two large liver lesions concerning for hepatic adenomas. When surveillance imaging showed a significant growth of the liver lesions, biopsy was pursued, which confirmed a diagnosis of hepatic adenomas. However, given the size of these lesions, resection was not a viable option for the patient. Instead, the patient underwent liver transplantation at age 41, which he tolerated well. Our case demonstrates the utility of deceased donor liver transplantation as a treatment for patients with Abernethy syndrome complicated by unresectable adenomas.

## Introduction

Congenital extrahepatic portosystemic shunts (CEPS) [1] bypass the normal intrahepatic vascular anatomy. Due to advances in imaging techniques, an increasing number of patients are being diagnosed with Abernethy syndrome in the perinatal period as antenatal ultrasound can identify abnormal portosystemic communication or an enlarged umbilical vein [2,3]. The current estimated incidence is one per 30,000 live births [4]. Several classification systems have attempted to describe this heterogeneous group of vascular malformations. The most used system, proposed by Morgan and Superina [5], divides cases into types 1 and 2. In type 1 Abernethy syndrome, there is complete agenesis of the intrahepatic portal system, and the splanchnic circulation forms an extrahepatic shunt with the systemic venous circulation. In type 1A, the superior mesenteric vein (SMV) and splenic vein (SV) drain individually into the IVC. In type 1B, the SMV and SV form a common portal vein, which drains into the inferior vena cava (IVC). In type 2 Abernathy syndrome, some intrahepatic portal flow is preserved, and there is only a partial extrahepatic shunt. Lautz et al. further differentiated type 2 into 2A and 2B [6]. In type 2A, either the right or left portal vein drains into the IVC. In type 2B, the primary portal vein drains into the IVC, but with the addition of a persistent connection to the liver. An alternative classification proposed by Kobayashi et al. defines Abernethy syndrome into three types depending on the location of the shunt [7]. In type A, the primary portal vein drains into the IVC. In Type B, the primary portal vein drains into the renal vein. In type C, the primary portal vein drains into the iliac vein. Focusing less on vascular anatomy and more on the degree of hepatic portal hypoplasia, Kanazawa et al. suggested splitting Abernethy syndrome into three types [8]. In type 1, there is only mild hypoplasia and intrahepatic portal flow is well-visualized on imaging. In type 2, there is moderate hypoplasia, and in type 3, there is significant or complete hypoplasia.

The development of Abernethy malformations is intimately related to embryological development. During embryological development, a failure of vitelline veins to anastomose with the hepatic sinusoids leads to the development of a type 1 Abernethy malformation. The location of the congenital portosystemic shunt with respect to the IVC is determined by which vitelline vein is affected; retrohepatic shunts are formed when there is a persistent right vitelline vein, while infra-hepatic shunts are formed when there is persistence of the left vitelline vein [3].

Abernethy syndrome has a broad spectrum of clinical manifestations. The median age at diagnosis is 21 years, with a range of 0 to 66 years [9]. The chronicity and severity of symptoms depend on the degree of intrahepatic portal vein hypoplasia and the volume of blood moving through any shunts that are present [4,10-11]. Patients diagnosed during childhood can have failure to thrive, psychomotor delay, cholestasis, and hypergalactosemia. Otherwise, the diagnosis is made incidentally when patients are worked up for abdominal pain, transaminitis, hepatopulmonary syndrome, portopulmonary hypertension, or portosystemic encephalopathy. Rarely, patients may present with lower gastrointestinal bleeding as a result of rectal varices in the case of a portosystemic shunt that drains portal blood into the iliac vein via an inferior mesenteric vein [3].

After being diagnosed with Abernethy syndrome, patients often seek medical care in the future for management and treatment of a hepatic mass that may be found during routine follow-up. Approximately half of patients with Abernethy syndrome eventually develop one or multiple hepatic adenomas, which may progress to hepatocellular carcinoma (HCC) [12]. Decreased hepatic portal blood supply secondary to a portosystemic shunt has been shown to increase hepatic artery blood flow, bringing more growth factors to the hepatic parenchyma [12]. Increased activation of molecular signaling pathways in response to these growth factors is thought to be responsible for the increased incidence of liver neoplasms in patients with vascular abnormalities like Abernethy syndrome [13,14].

The management of Abernethy syndrome depends on which type the patient would be classified as any associated complications they have experienced and associated anomalies. Type 1 is generally treated with liver transplantation, as patients are more likely to develop life-threatening progression of hepatopulmonary syndrome [11,15]. Type 2 can be treated by shunt closure, which can be done in a primarily interventional manner or in a two-step approach wherein the shunt is first banded surgically and then closed interventionally a few months later. For patients with type 2 Abernethy syndrome, all symptomatic patients must be treated with shunt closure. However, asymptomatic patients should be treated according to the shunt ratio, which, if higher, correlates with an increased risk of hepatic encephalopathy, thus warranting treatment with shunt closure [16]. 

Any hepatic lesions discovered on follow-up should be further characterized with a triple-phase MRI study aided with agents, such as gadobenate dimeglumine or gadoxetic acid. These agents can help diagnose focal nodular hyperplasia (FNH) or HCC without the need for biopsy [3]. For lesions concerning for HCC, management is highly dependent on the stage of the disease. Early-stage tumors may be cured with resection or liver transplantation if Milan criteria are met. Intermediate-stage tumors should be treated with locoregional therapies, such as embolization. Patients with advanced-stage disease should receive systemic chemotherapy [17]. For lesions suspected to be hepatic adenomas, management is dependent on the lesion size, pattern of progression, and patient sex. In female patients with adenomas less than 5 cm, a six-month period of observation with lifestyle changes, such as cessation of oral contraception, can be attempted. Continued growth is an indication for surgery [18]. Surgery is always indicated in male patients with adenomas regardless of size [19].

For patients with unresectable hepatic adenomas, a liver transplant is the only curative treatment [20]. To our knowledge, our report is the first case reported in the literature where a patient with unresectable adenoma, containing focal HCC, in the setting of Abernethy syndrome received deceased-donor orthotopic liver transplantation (OLT).

## Case presentation

This 41-year-old male patient was diagnosed at birth with vertebral abnormalities, anal atresia, esophageal atresia, a cardiac defect, and a solitary right kidney (VATER syndrome). The patient did not have an associated tracheoesophageal fistula. The patient also had a history of seizure disorder and a cerebrovascular accident that occurred shortly after birth, resulting in persistent left-sided weakness. At the age of five, the patient was found to have multiple hepatic lesions and subsequently underwent partial hepatectomy for the resection of the masses. Final pathology demonstrated focal nodular hyperplasia and hemangioma, but no adenoma. During his preoperative evaluation at the time, the patient was found to have Abernethy syndrome (type 1b). His SMV and SV combined into a primary portal vein, which drained directly into his infrahepatic IVC at the liver hilum. Consistent with type 1B, no portal venous blood flow directly into the liver was noted.

At age 33, approximately eight years prior to transplantation, during a routine outpatient hepatology appointment, the patient was found to have a new mild elevation in aspartate aminotransferase (AST) to 50 U/L and a significant elevation in alkaline phosphatase to 269 U/L. He was otherwise asymptomatic and without any pertinent findings on physical exam.

**Figure 1:**
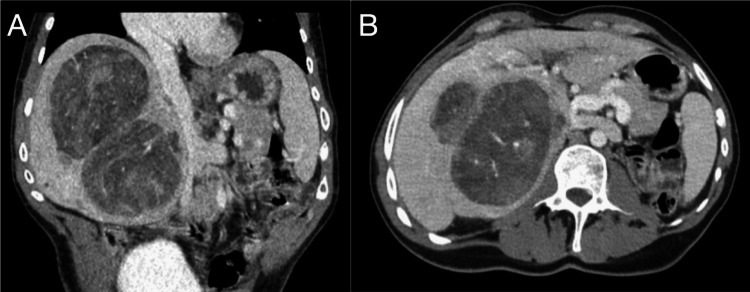
Pre-transplantation CT demonstrating hepatic lesions and type 1B Abernathy syndrome (A-B) Two dominant lesions noted in the left liver, measuring 9.3 x 8.6 cm and 10.8 x 7.9 cm. Several smaller lesions were also noted in the left liver. Delayed images demonstrated portal vein (PV) anatomy. Direct drainage of the PV into the infra-hepatic inferior vena cava (IVC) was noted in (A) axial and (B) coronal views.

Given the patient’s history of Abernethy syndrome and hepatectomy, computed tomography (CT) imaging was performed, which revealed two new liver lesions that were consistent with hepatic adenomas. At that time, the lesions measured 6 cm x 4 cm and 4.6 cm x 4.2 cm with minimal biliary dilation. The patient was asymptomatic, so he was surveilled with regular imaging and follow-up appointments for the next five years. Three years prior to surgery, surveillance images showed a significant growth of the hepatic tumors, with the largest lesion being 8 cm in diameter. CT-guided core biopsy of the 8 cm lesion confirmed the diagnosis of hepatic adenoma, with immunohistochemistry demonstrating it to be of the β-catenin-activated subtype. Biopsy of the background liver demonstrated the absence of portal vein profiles in portal tracts, consistent with Abernethy syndrome (type 1). The patient had persistent elevations in serum liver transaminase levels, with an AST of 57 and an alkaline phosphatase of 305, but the patient continued to be asymptomatic. During subsequent surveillance imaging, the lesions (Figure 1) measured 9.3 cm x 8.6 cm and 10.8 cm x 7.9 cm, spanning the majority of the patient’s remnant liver without any vascular involvement, pressure effect, or invasion into other organs. Considering the patient's history of prior liver resection and significantly sized lesions, his disease was deemed unresectable despite the fact that adenoma resection is the treatment of choice for male patients with adenomas. Given the size, growth behavior, and multiplicity of the tumors, the patient was listed for liver transplantation.

He was granted UNOS (United Network for Organ Sharing) exception points, for adenoma with a risk of malignant transformation and unresectable adenoma, after our team submitted a request to the National Liver Review Board. At the age of 41, the patient underwent deceased donor OLT from a 25-year-old female brain-dead donor who died of an anoxic injury. After full mobilization of the native liver, porta-hepatis dissection confirmed the preoperative diagnosis of type 1b Abernathy syndrome where the recipient's SMV and SV combined into a portal vein that drained directly into the infrahepatic IVC. Further dissection revealed standard arterial anatomy. Hepatic arteries were controlled, and the aberrant portosystemic shunt was stapled off after clamping the portal vein to be used for portal perfusion of the graft. Native liver hepatectomy was completed. After back-table preparation, the liver was transplanted using a piggyback technique with side-to-side cavocavostomy. Standard end-to-end porto-portal reconstruction was performed using the recipient's native portal vein stump. The liver graft was re-perfused with no major concerns. Arterial and biliary reconstructions were then performed in the usual fashion where an end-end arterial anastomosis and a duct-duct biliary reconstruction without stenting were performed, respectively.

**Figure 2:**
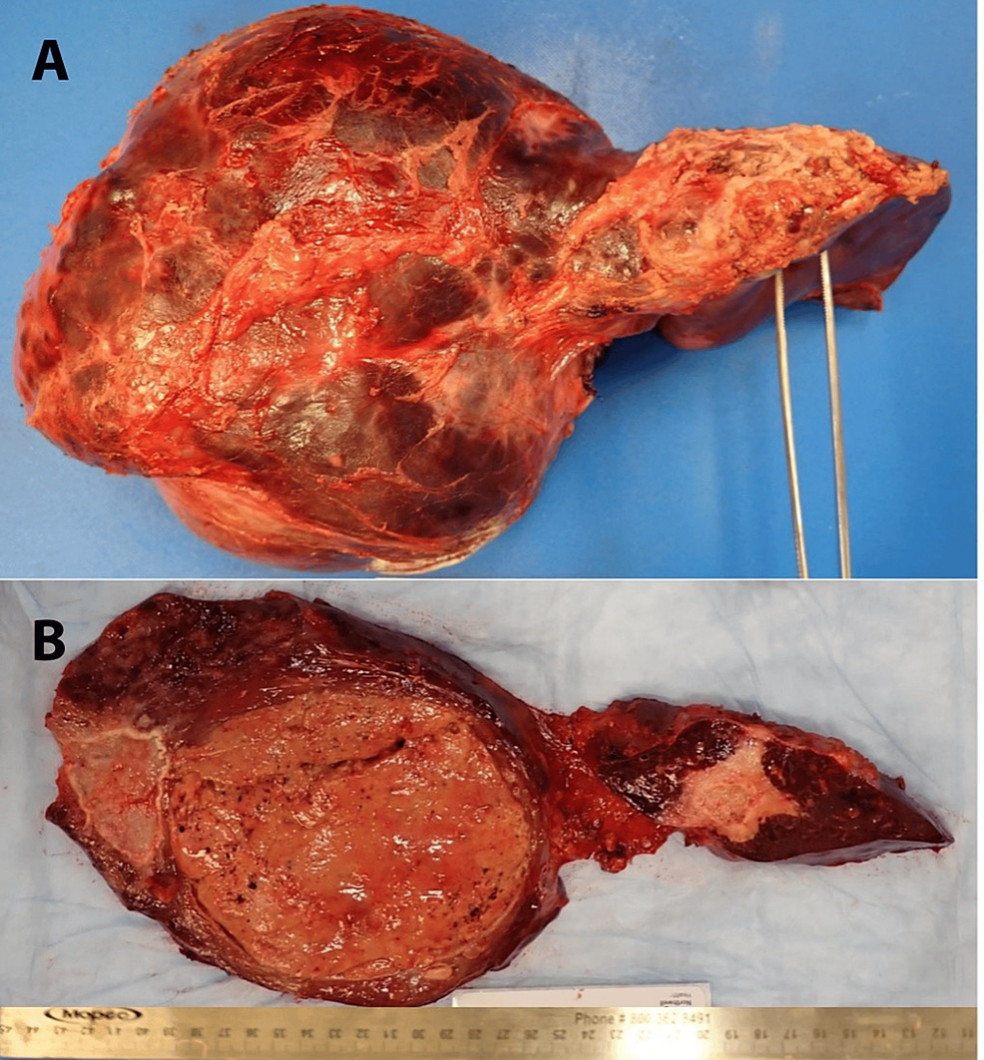
Gross images of the resected native liver (A) Inferior view, showing a massively enlarged right lobe (left side of the image), with a small residual left lobe (right side of the image). (B) Horizontally sectioned liver, showing an 11.5 cm dominant tumor in the right lobe, a lateral 5.2 cm tumor, and a smaller left lobe with a central scar.

**Figure 3:**
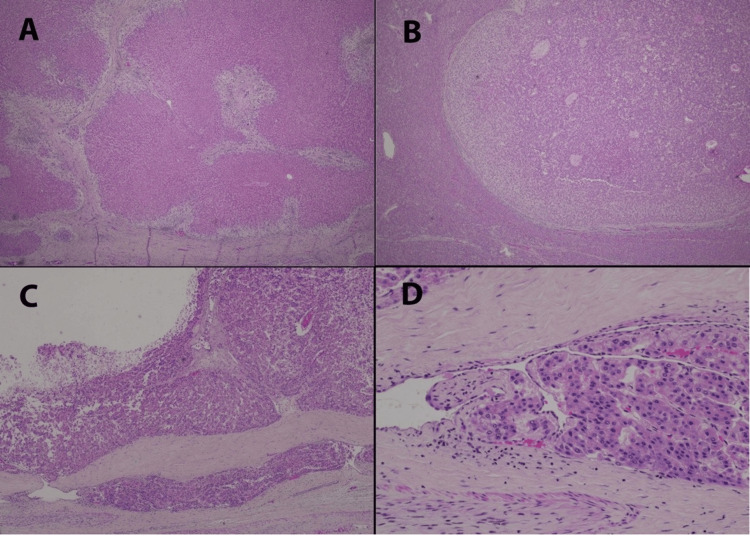
Histological images of the explanted liver pathology (A) Explanted liver, background liver tissue, showing extensive geographic fibrosis in broad fibrous septae, which lack portal vein profiles. (B) Low power view of a portion of the right lobe 11.5 cm tumor, showing the monotonous features of hepatocellular adenoma. (C) Medium power of one peripheral region of the 11.5 cm lesion, showing well-differentiated hepatocellular carcinoma with invasion into the peritumoral vasculature (bottom of the image). (D) High power of the region of vascular invasion, showing trabecular architecture characteristic of hepatocellular carcinoma. Hematoxylin-and-eosin stained sections, at 25X (A), 40X (B), 100X (C), and 400X (D).

The resected specimen was sent to the pathology department for histopathology examination. Upon gross examination, the resected liver weighed 2066 g. An inferior view of the intact liver showed a massively enlarged right lobe, and a much smaller residual left lobe (Figure 2A). The horizontally sectioned liver, in the same orientation, shows that the right lobe of the liver was substantively replaced by a dominant tumor 11.5 cm in maximum diameter, with a smaller 5.2 tumor mass laterally; the left lobe contained a large scar, consistent with prior surgery (Figure 2B). Microscopic examination showed extensive geographic fibrosis throughout the background liver (Figure 3A); portal vein profiles could not be identified. There were numerous regenerative nodules of benign hepatocellular parenchyma, 1.5 cm or less in diameter. The dominant right lobe tumor was hepatocellular adenoma (Figure 3B). However, a focal region exhibited well-differentiated hepatocellular carcinoma, invasive into the peritumoral vasculature (Figure 3C, 3D). The 5.2 cm lateral tumor was found to be a sclerosed hemangioma, with entrapped foci of mature adipose tissue exhibiting dystrophic calcification along their periphery. This histology was consistent with a hamartoma that had undergone extensive sclerosis and focal calcification. Immunohistochemistry of the 11.5 cm lesion demonstrated nuclear positivity for $\beta$-catenin in the focus of hepatocellular carcinoma; the adenoma was otherwise negative for $\beta$-catenin nuclear staining. Molecular analysis of the tissue block containing hepatocellular carcinoma demonstrated microsatellite status to be stable; tumor mutational burden to be low (three mutations per Mb), with four genomic mutations identified: BARD1 V523I, CTNNB1 K335I, SGK1 M1T, and TERT promoter 124C>T. Immunohistochemistry for PDL1 was negative. 

**Figure 4:**
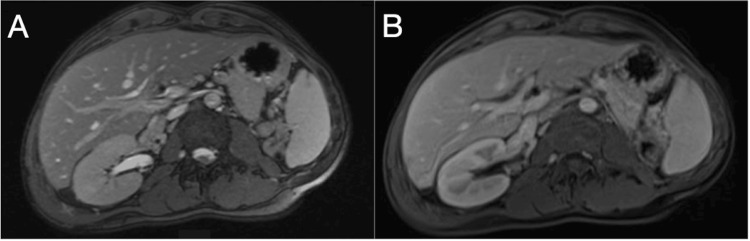
Post-transplantation MRI (A-B) Post-transplant graft with a normal-appearing parenchyma.

The patient’s immediate post-transplant course was uncomplicated, and liver ultrasound showed patent vasculature with a good hepatopetal flow and normal flow hemodynamics. On postoperative day 10, he was discharged to a subacute rehabilitation facility and then to home. Magnetic resonance imaging (MRI) performed one year post-transplantation demonstrated a healthy hepatic graft (Figure 4). Eighteen months post-transplantation, the patient continues to have good liver graft function on a stable anti-rejection medication regimen of mycophenolate mofetil, low-dose prednisone, and tacrolimus.

## Discussion

**Figure 5:**
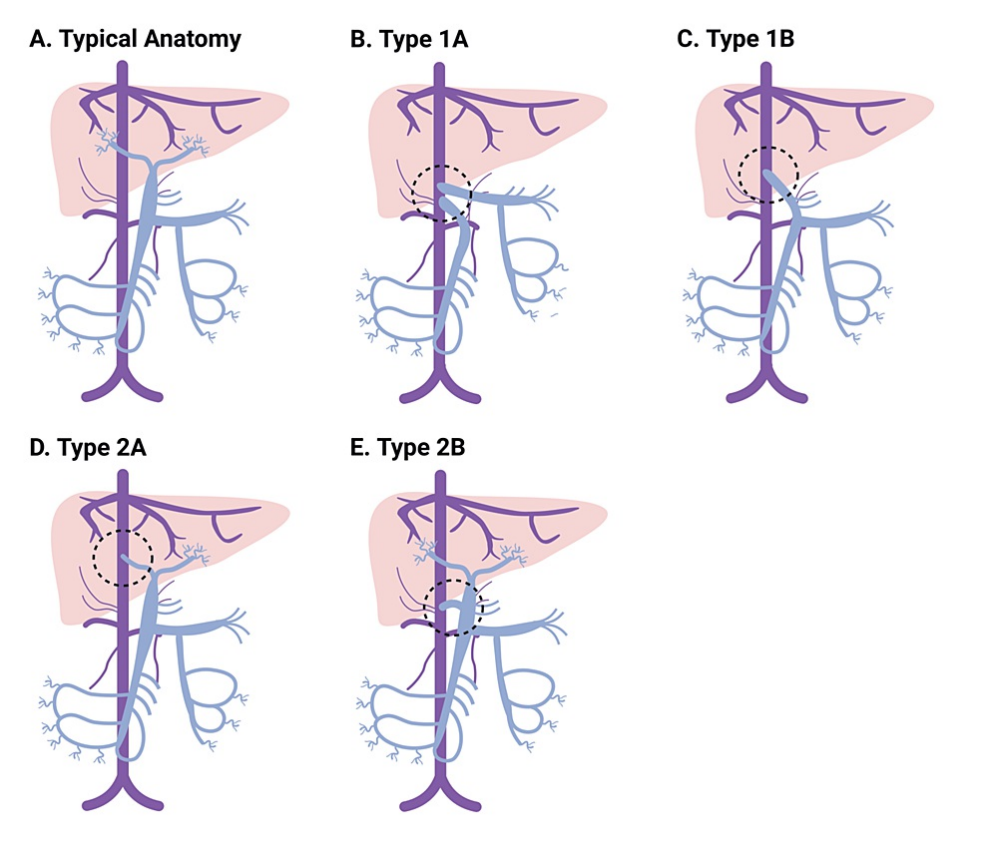
Variable congenital portosystemic shunts in Abernethy syndrome (A) Normal portal and systemic venous anatomy. The most commonly used system for defining Abernathy syndrome splits anatomy into two types. In type 1, there is complete agenesis of the intrahepatic portal veins. (B) In type 1A, the superior mesenteric vein (SMV) and splenic vein (SV) drain separately into a systemic vein. (C) In type 1B, the SMV and SV for a primary portal vein (PV), which drains into a systemic vein. In type 2, there is some retained intrahepatic portal flow, although there is hypoplasia. (D) In type 2A, either the right or the left PV drains into a systemic vein. (E) In type 2B, there is an extrahepatic shunt between the PV and a systemic vein, with preservation of some distal PV structures. Image Credits: Dane Thompson MD, PhD

Here, we describe a case of OLT as a treatment for a patient with Abernethy syndrome (Figure 5A-5E) and unresectable hepatic adenoma. This is the second reported case of OLT for this indication in a patient with Abernethy syndrome. Brasoveanu et al. previously described a case of a young woman in her 20s with type 1B Abernethy syndrome and unresectable hepatic adenomas treated with living-donor, partial OLT. The patient was largely asymptomatic in the pre-transplant period. She did not present with any signs of hepatic encephalopathy, hepatopulmonary syndrome, or liver failure. She first developed a large adenoma in the right hepatic lobe but subsequently developed another large adenoma in the left hepatic lobe and determined to be unresectable. Due to the progression of the disease while on the transplant waiting list, and because of the low likelihood of a deceased-donor transplant owing to the patient’s stable liver function, the patient’s treatment team decided to pursue a living-donor transplant [21]. To our knowledge, our report is the first case where a patient with unresectable adenoma, containing focal HCC, in the setting of Abernethy syndrome received a deceased-donor OLT.

To date, there have been roughly 30 overall cases of Abernethy syndrome-related complications treated with liver transplantation. These cases included pediatric and adult patients presenting with a wide variety of symptoms (Table 1). Most patients, regardless of age, had successful outcomes for months to years after transplant [21].

**Table 1:** Summary of published cases of complications related to Abernethy syndrome treated with liver transplantation

Authors	Abernathy syndrome type	Indication for transplant	Transplant type	Duration of follow-up and outcome
Barton JW et al., 1989 [22]	1a	Hepatoblastoma	OLT	Stable at 18 months
Woodle ES et al., 1990 [23]	1a	Biliary atresia	OLT	Stable at 9 months
Morgan G et al., 1994 [5]	1b	Biliary atresia	Split-liver transplant	Expiration after transplant due to bowel necrosis
Howard ER et al., 1997 [24]	1b	Biliary atresia	OLT	Not mentioned
Taoube KA et al., 1999 [25]	Not reported	Biliary atresia	OLT	Not mentioned
Andreani et al., 2000 [26]	1b	Biliary atresia	OLT (reduced size)	Stable at 20 months
Shinkai M et al., 2001 [27]	1	Portosystemic encephalopathy	OLT	Not mentioned
Charre L et al., 2004 [28]	1	Recurrent hematochezia complicated by incapacitating anemia	OLT	Not mentioned
Wojcicki M et al., 2004 [29]	1a	Portosystemic encephalopathy	OLT	Stable at 2.5 years
Ohnishi Y et al., 2005 [30]	SMV draining into the azygous vein	Intrapulmonary shunt and brain abscesses	Living donor reduced-size liver transplant	Stable at 5 months
Takeichi T et al., 2005 [31]	1b	Portosystemic encephalopathy	Domino liver transplant	Stable at 10 months
Soejima Y et al., 2006 [32]	1b	Persistent hyperammonemia, hypergalactosemia	Living donor (left lateral section) transplant, with preservation of the recipient’s native right hepatic lobe	Stable at more than 3 months post transplant
Sumida W. et al., 2006 [33]	1a	Portosystemic encephalopathy	Living donor liver transplant	Stable at 19 months
Witters et al., 2007 [34]	2	Hepatocellular carcinoma	OLT	Not mentioned
Emre S et al., 2007 [35]	1b	Hepatopulmonary syndrome	Partial orthotopic liver transplant from deceased donor	Stable at 15 months
Elias et al., 2008 [15]	1b	Hepatopulmonary syndrome	OLT	Stable at 5 months
Singhal A et al., 2009 [36]	1b	Biliary atresia, hepatopulmonary syndrome, and hepatic nodules	Living donor liver transplant (left hepatic lobe)	Stable at 18 months
Kasahara M et al., 2009 [37]	Portosystemic shunt between SMV and splenic vein and R renal vein	Recurrent hyperammonemia	Living donor liver transplant (left lateral segment)	Stable
Matsuura T et al., 2010 [38]	Portosystemic shunt between SMV and Right internal iliac vein via IMV	Recurrent hyperammonemia	Liver transplant (extended left hepatic lobe) from a living donor	Stable at 3 months
Hori T et al., 2010 [39]	1b	Pulmonary hypertension	Living donor liver transplant of extended left lateral segment	Stable
Osorio MJ et al., 2011 [40]	1b	Hepatopulmonary syndrome with unresectable FNH	OLT	Stable at 6 months
Law YM et al., 2011 [41]	Portosystemic shunt between convergence of SMV and splenic and azygous veins	Severe pulmonary hypertension	Split liver transplant of left lateral segment	Post-transplant course complicated by intrahepatic biliary strictures requiring transplantation and chronic graft rejection with eventual death 2 years post retransplantation
Uchida et al., 2012 [42]	1a	Hepatopulmonary syndrome	Living donor transplant of left hepatic lobe	Stable at 3 year follow-up
Gordon-Burroughs et al., 2014 [43]	1b	Recurrent HCC post left hemihepatectomy	OLT	Stable and without recurrent HCC at 3 years
Brasoveanu et al., 2015 [21]	1b	Unresectable liver adenomatosis	Living donor transplant of left lateral segment	Stable at 9 months
Nam H. et al., 2020 [44]	1b	Recurrent hyperammonemia	Living donor transplant of right hepatic lobe	Stable at 1 year
Present case	1b	Unresectable liver adenomatosis	OLT	Stable at 18 months

In patients with Abernethy malformations, increased hepatic artery blood flow to the parenchyma occurs due to loss of portal perfusion. This in turn leads to growth factor-mediated activation of molecular signaling pathways that have been implicated in the pathophysiology of hepatocellular adenomas [14]. These tumors have been subdivided into five types depending on genetic sequencing: hepatocyte nuclear factor-1 alpha (HNF1) mutated adenomas, β-catenin mutated adenomas, inflammatory/telangiectatic adenomas (IL-6 signal transducer gene mutated), sonic hedgehog mutated adenomas, and unclassified type. Of these subtypes, the β-catenin mutated adenomas carry the highest risk of malignant conversion. This was indeed the case in this patient, with the CT-guided core biopsy of what was then the dominant 8 cm lesion showing β-catenin nuclear immunoreactivity, indicative of β-catenin activation. Patients with biopsy evidence of this type should be managed per the National Comprehensive Cancer Network (NCCN) guidelines for patients with hepatocellular carcinoma [13]. In the case of patients like ours who underwent resection, these guidelines suggest that these patients should undergo high-quality, multiphasic, CT imaging of the chest, abdomen, and pelvis and serum testing for alpha fetal protein (AFP) every three to six months for the first two years and then every six months thereafter [45].

From the liver explant, the CTNNB1 K335I finding in the region of the 11.5 cm adenoma containing hepatocellular carcinoma explant confirms the presence of an activating mutation of the β-catenin gene. CTNNB1 is the most frequently mutated gene in hepatocellular carcinoma [46]. BARD1 mutation is reported in 1.1% of hepatocellular adenomas, SGK1 mutation is observed in numerous tumor types, and TERT is also one of the most commonly mutated genes in hepatocellular carcinoma, with mutations predominantly in the reporter region that regulates gene expression, as in this case [47].

## Conclusions

There are no established guidelines for managing patients with Abernethy syndrome that have hepatic adenomas. However, these lesions have a propensity to grow and may become symptomatic or harbor focal malignancy as we saw in this case. Future work should be done to establish evidence-based guidelines for the management of hepatic adenomas in patients with Abernethy syndrome as, in recent years, the literature has reported an increasing number of cases involving patients with this rare malformation.

Patients diagnosed with Abernethy syndrome at birth or early in life undergo radiological screenings that may identify hepatic lesions in their early stages. For patients with resectable lesions that are larger than 5 cm, a partial hepatectomy should be pursued. If the disease is unresectable, a liver transplant should be pursued as it offers the patient treatment of not only their liver lesions but also correction of their Abernethy malformation.
